# Imidazole, a New Tunable Reagent for Producing Nanocellulose, Part I: Xylan-Coated CNCs and CNFs

**DOI:** 10.3390/polym9100473

**Published:** 2017-09-27

**Authors:** Jia Mao, Hatem Abushammala, Hubert Hettegger, Thomas Rosenau, Marie-Pierre Laborie

**Affiliations:** 1 Chair of Forest Biomaterials, Faculty of Environment and Natural Resources, Albert-Ludwig-University of Freiburg, Werthmannstr 6, 79085 Freiburg, Germany; jia.mao@biomat.uni-freiburg.de (J.M.); habushammala@gmail.com (H.A.); 2 FIT-Freiburg Center for Interactive Materials and Bioinspired Technologies, University of Freiburg, Georges-Köhler-Allee 105, 79110 Freiburg, Germany; 3 Division of Chemistry of Renewables Resources, Department of Chemistry, University of Natural Resources and Life Sciences Vienna, Muthgasse 18, 1190 Vienna, Austria; hubert.hettegger@boku.ac.at (H.H.); thomas.rosenau@boku.ac.at (T.R.)

**Keywords:** imidazole, cellulose nanocrystals, cellulose nanofibrils, xylan

## Abstract

Imidazole is reported to be an effective reactant for the production of nanocellulose from hardwood pulp. The morphologies and surface properties of the nanocellulose can be simply tailored according to the water content in the imidazole system: with pure imidazole, cellulose nanofibrils (CNFs) in a yield of 10 wt % can be produced. With 25 wt % of water in imidazole, cellulose nanocrystals (CNCs) are obtained in 20 wt % yield. Both nanocelluloses exhibit crystallinity indices in the order of 70%. Interestingly, they retain the original xylan from the pulp with ca. 9–10 wt % of residual xylan content.

## 1. Introduction

Nanocellulose is a promising bio-based building block for material design. It is conventionally produced by acid hydrolysis or mechanical fibrillation to yield cellulose nanocrystals (CNCs) or cellulose nanofibrils (CNFs), respectively [1,2,3,4,5,6]. While CNCs are rod-like nanoparticles of high aspect ratio, CNFs display an entangled, networked morphology. Both types of nanocellulose exhibit one nanoscale dimension coupled with high crystallinity indices (80–95%), which contribute to their high mechanical and unique optical properties. As a result, production and commercialization of nanocellulose-enabled materials is expected to grow in the medium term [7,8]. 

CNC production with conventional Brønsted acids was first reported to occur in rather low yields of 20–30 wt % [9]. Subsequently, many endeavors have examined alternative production methods with a view to improve material, environmental and economical efficiencies [10]. Both physico-mechanical processes, such as ultrasonication [11], homogenization [12], autoclaving [13], and chemical swelling/oxidation pretreatments [14,15,16], have been designed to improve the accessibility to cellulose, facilitating further acid-attack for efficient nanocrystal production. 

Among the approaches used to extract CNCs/CNFs, imidazolium-based ionic liquids (ILs) have been particularly efficient, as reported by Man et al. and others [17,18,19]. In these systems, a protic anion, HSO_4_^‒^, was proposed to catalyze the hydrolysis of cellulose, leading to the liberation of CNCs. The imidazolium cation was not ascribed any particular activity in these studies [17,18,19]. However, a recent study with imidazolium-based ionic liquids revealed that side reactions stemming from imidazolium were also possible on cellulose [20]. In particular, it was shown that imidazole and 1-acetylimidazole, both thermal degradation products of 1,3-dialkylimidazolium acetate, play a key role in acetyl transfer towards cellulose. Previously, it has already been ascertained that C-2 of 1,3-dialkylimidazolium cations also reacts with the reducing ends of cellulose [21,22].

The activity of imidazole on cellulose demonstrated in these previous studies motivated our investigation on the possible action of imidazole alone on cellulose pulp. Imidazole is an amphoteric compound due to its two nitrogens: N-1 and N-3. It is base-dominant with a p*K*_b_ of 7 and p*K*_a_ of 14. It is an aromatic, planar molecule with six pi electrons and prone to self-association in dilute aqueous solutions by vertical stacking and H-bond formation [23]. Imidazole has similar characteristics to ILs such as negligible vapor pressure, making it easy to handle and recover [24]; however, the price of imidazole is very competitive (0.2 Euro/g vs. 1–6 Euro/g) compared to ionic liquids [25]. Imidazole has been extensively used in industry as a corrosion inhibitor of transition metals, such as copper [26]. Its potential in biomass fractionation has also been recently demonstrated [24,27]. In these studies, imidazole proved to be an efficient solvent for delignifying biomass even under relatively moderate conditions [24,27]. 

To the best of our knowledge, attempts to process pulp with imidazole have not been reported. In this work, we demonstrate the potential of imidazole on processing pulp towards nanocellulose. The role of the imidazole’s water content on the type and morphology of the resulting nanocellulose is also presented. 

## 2. Materials and Methods

### 2.1. Materials

Bleached hardwood (Birch, Betula) Kraft pulp was provided by Stora Enso (Imatra Mills, Finland). ca. 12% of xylose was contained in addition to cellulose. Imidazole was purchased from Sigma Aldrich. Imidazole/water mixtures with different water contents of 0, 5, 25, 50 and 75 wt % were prepared using an analytical balance (Sartorius, Mettler Toledo, Giessen, Germany) with a precision of 0.01 mg. 

### 2.2. Treatment of Hardwood Pulp in Imidazole/Water Mixture

**Figure 1:**
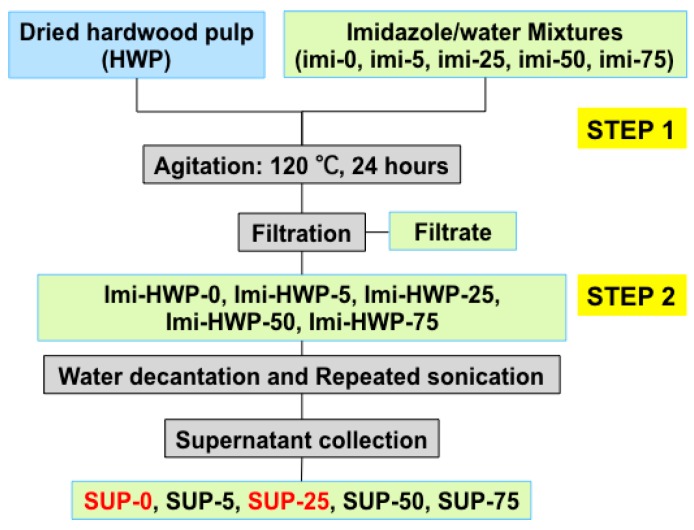
Two-step treatment of hardwood pulp in imidazole/water mixtures.

In the first step, 300 mg of freeze-dried bleached hardwood Kraft pulp (HWP) was mixed with an aqueous imidazole mixture pre-heated at 120 °C. A set of aqueous imidazole mixtures was used with the following water contents: 0, 5, 25, 50 and 75 wt %, which are referred to as imi-0, imi-5, imi-25, imi-50 and imi-75, respectively (Figure 1). The solid to liquid ratio (pulp to imidazole/water) was kept constant at 1:30 (*w*/*w*). The mixture was mechanically agitated under reflux at 120 °C for 24 h. After agitation, the reaction mixture was quenched by adding 25 g of water; the mixture was then filtered (nylon filter membranes, pore size: 0.22 μm, Sigma Aldrich, Germany) to collect the treated pulp. It was extensively washed to remove any dissolved cellulose fragments and residual imidazole. The treated pulp samples are thereafter referred as Imi-HWP-0, -5, -25, -50 and -75 (Figure 1). 

Mass loss (ML) upon this treatment was calculated from three batches using the oven-dried mass of the original pulp (*M*_o_ (g)) and of the solid pulp residue collected after treatment (*M* (g)):

(1) $$\mathrm{ML}\;\left(\%\right)=\frac{M_{o}-M}{M_{o}}\;\times 100\%$$

In a second step, each of the treated pulps was dispersed in water (ca. 10 mg/mL) and sonicated at a power of 60% for 1 min (Power 100 W, Sonopuls HD 3200, Omnilab, Berlin, Germany). An ice bath was used during the sonication to avoid overheating. The turbid supernatant of the treated pulp dispersions was collected after standing for one hour. More turbid supernatants were obtained after adding water of the same volume followed by sonication. This decantation process was repeated (around ten times) until no further turbid supernatant was formed. The collected supernatants are named as SUP-0, -5, -25, -50 and -75 (Figure 1). Soxhlet extraction against hot water was applied to further purify the samples. SUP-0 and SUP-25 suspensions were mainly chosen for further characterization.

### 2.3. Characterization of the Original Pulp (HWP), Treated Pulps (Imi-HWP) and Collected Supernatants (SUP)

#### 2.3.1. HWP and imi-HWP Characterization

**Gel Permeation Chromatography (GPC) Analysis.** The freeze-dried HWP, treated pulps (Imi-HWP) and collected supernatants (SUP) were characterized for number-average degree of polymerization (DP_n_), carboxyl (–COOH) and carbonyl (–C=O) content according to the well-known FDAM and CCOA methods [28,29,30,31,32]. A modified Kontron 420 pump (Tegimenta AG, Rotkreuz, Switzerland) GPC system equipped with four serial columns (PLgel MIXED-A LS, 20 μm, 7.5 mm × 300 mm, Agilent Technologies, Santa Clara, CA, USA), ), and fluorescence (TSP FL2000, Thermo Separation Products, Waltham, MA, USA), MALLS (Wyatt Dawn DSP, Wyatt Technology Corporation, Santa Barbara, CA, USA), and RI (Shodex RI-71, Showa Denko, Munich, Germany) detectors was used. Eluent: DMAc/LiCl (0.95%, *w*/*v*), Flow rate: 1.00 mL/min, Temperature: 25 °C, Injection volume: 200 μL, Runtime: 45 min. A refractive index increment of 0.140 mL/g was used. For carbonyl content, the cellulose samples were treated with the carbonyl-selective fluorescence label, carbazole-9-carbonyloxyamine (CCOA).

**Methanolysis for Compositional Analysis.** Acid methanolysis followed by GC/MS analysis was performed according to a method adapted from Sundberg et al. [33]. GC-MS analysis was performed on an Agilent 6890N GC and an Agilent 5975B inert XL MSD quadrupole mass-selective detector (EI: 70 eV), using an Agilent HP-5MS capillary column (30 m × 0.25 mm i.d.; 0.25 μm film thickness, all Agilent Technologies, Santa Clara, CA, USA) and helium as the carrier gas with a pressure of 0.94 bar, a flow rate of 1.1 mL min^−1^, a split flow rate of 7.5 mL·min^−1^, and a split ratio of 7:1.

#### 2.3.2. SUP Characterization

**Wide-angle X-ray Diffraction (WAXD).** WAXD measurements were carried out on the original HWP and the freeze-dried turbid suspensions (SUP-0 and SUP-25). All samples were filled in a brass sample holder without applying any pressure. X-ray diffraction curves were recorded in the reflection mode in the 2θ-range of 5°–40° with a resolution of 0.01° using a 3003TT X-ray powder diffractometer (Seifert, Ahrensburg, Germany) with CuKα radiation (λ = 0.15405 nm).

The cellulose crystallinity index (*CrI*) was calculated from the crystalline (*I*_200_) and amorphous (*I*_am_) signal intensities at 2θ ≈ 22.1° and at 2θ ≈ 18°, respectively [34]:

(2) $$CrI=\frac{I_{200}-\;I_{\mathrm{am}}}{I_{200}}\times 100$$

Coherence lengths of the cellulose crystallites were derived for two directions: crystal thickness τ with [200] reflection at 2θ ≈ 22.1°, length B with [004] reflection at 2θ ≈ 34.5° applying the Scherrer equation [35]:

(3) $$\tau /B=\frac{K\lambda }{\beta \cos\theta }$$

where K is the shape factor 0.94, λ is the wavelength (0.15405 nm), β is the integral line breadth estimated from the peak width decomposed with the Gaussian representing [200], [004] reflection, and 2θ is the Bragg angle of the reflection.

**Atomic force microscopy (AFM).** A droplet of the dilute turbid suspensions (SUP-0 and SUP-25) after sonication (60% power, 1 min) was dried overnight on freshly cleaved mica. The topography of the samples was observed in the tapping mode on an AFM (Nanoscope III) equipped with a tube scanner from Digital Instruments (Veeco, Santa Barbara, CA, USA) using silicon tips (PPP-NCH, Nanoandmore, Wetzlar, Germany) with resonance frequency and spring constant of 360 kHz and 50 N/m, respectively. The height images were analyzed with Nanoscope Analysis 1.4 software (Brucker, Camarillo, CA, USA) for the determination of the particle dimensions.

**Zeta Potential (ζ).** To further characterize the surface properties of HWP and the extracted turbid suspensions (SUP-0 and -25), zeta potential (ζ) was determined by a zeta sizer (Malvern Instrument, Herrenberg Germany). All samples were diluted to 0.05 wt % with a pH ≈ 6, and ζ was measured and reported as averages of at least three runs.

## 3. Results and Discussion

### 3.1. Process Characterization

**Figure 2:**
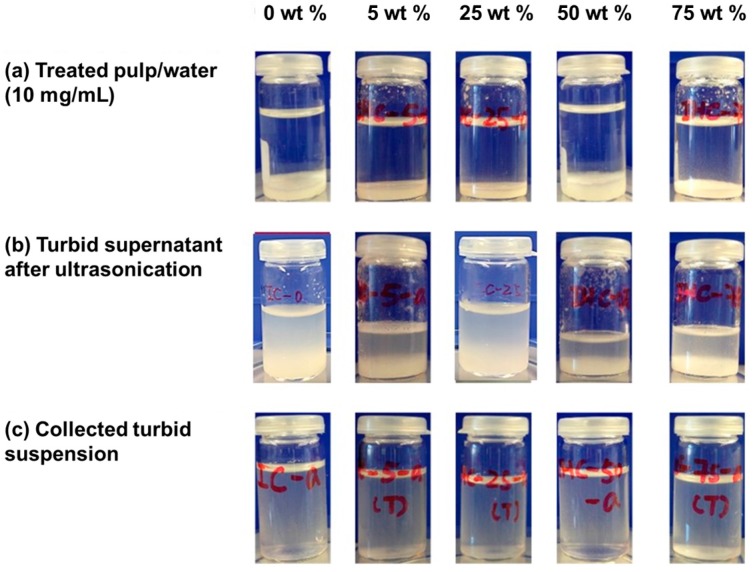
Dispersions of the treated pulps (imi-HWP) 0, 5, 25, 50 and 75 wt % represent the water contents of the used imidazole/water systems) (**a**); dispersions after sonication at a power level of 60% for 1 min (**b**); and the dilute turbid suspensions (SUP) (0.1 wt %) after standing for one week (**c**).

Treatment of bleached hardwood pulp with pure and aqueous imidazole mixtures at 120 °C for 24 h yields a solid residue (Figure 2a), which upon 1 min of sonication generates a turbid supernatant suspension (Figure 2b). Such turbid suspensions are generally associated with the presence of cellulose nanoparticles. The imidazole concentration in the aqueous solutions impacts significantly the volume of turbid fraction collected (Figure 2b) with the highest yields obtained from pure imidazole imi-0 (10 wt % yield) and from the imidazole aqueous mixture imi-25 (20 wt % yield). The latter concentration is similar to that previously reported for the optimization of CNC production from pulp with [Bmim]HSO_4_ [18]. Also, note that all turbid suspensions, when diluted to 0.1 wt %, remain stable for more than one week (Figure 2c).

**Figure 3:**
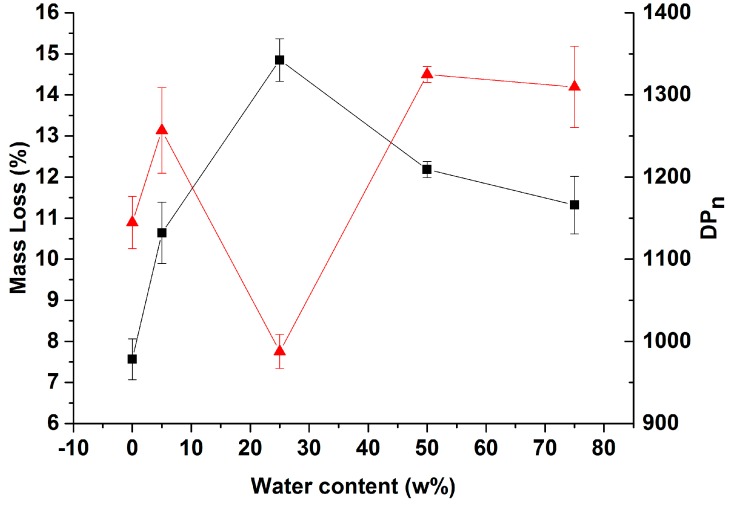
Averaged mass loss (black square) and number-average degree of polymerization (DP_n_) (red triangle) of the treated hardwood pulps (imi-HWP) (*n* = 3) in imidazole/water mixtures with different water contents at 120 °C for 24 h.

The water content of imidazole had a major impact on its action on cellulose. This can be preliminarily verified by monitoring mass loss upon the process and the degree of polymerization of the treated pulp as a function of the imidazole’s water content (Figure 3). When imidazole has a water content of 25 wt %, its ability to degrade cellulose pulp increases as evidenced by the steady increase with a maximum mass loss of 15% (Figure 3). Further dilution of imidazole results in less efficient degradation. The degradation activity of the imidazole-water mixtures can be considered from the DP_n_ behavior as a function of imidazole dilution (Figure 3). The DP_n_/water content curve is the mirror image of the mass loss trend (Figure 3). A minimum DP_n_ of 950 is measured when treating pulp with the 25-imi system, with which the maximum mass loss was also obtained. Below and above this concentration, degradation is less efficient and cellulose products retain a DP_n_ in the 1100–1300 range. Trends of mass loss and cellulose DP_n_ as a function of imidazole concentration reveal that cellulose cleavage kinetics can be tuned by the water content in imidazole.

### 3.2. Product Morphological Characterization

**Figure 4:**
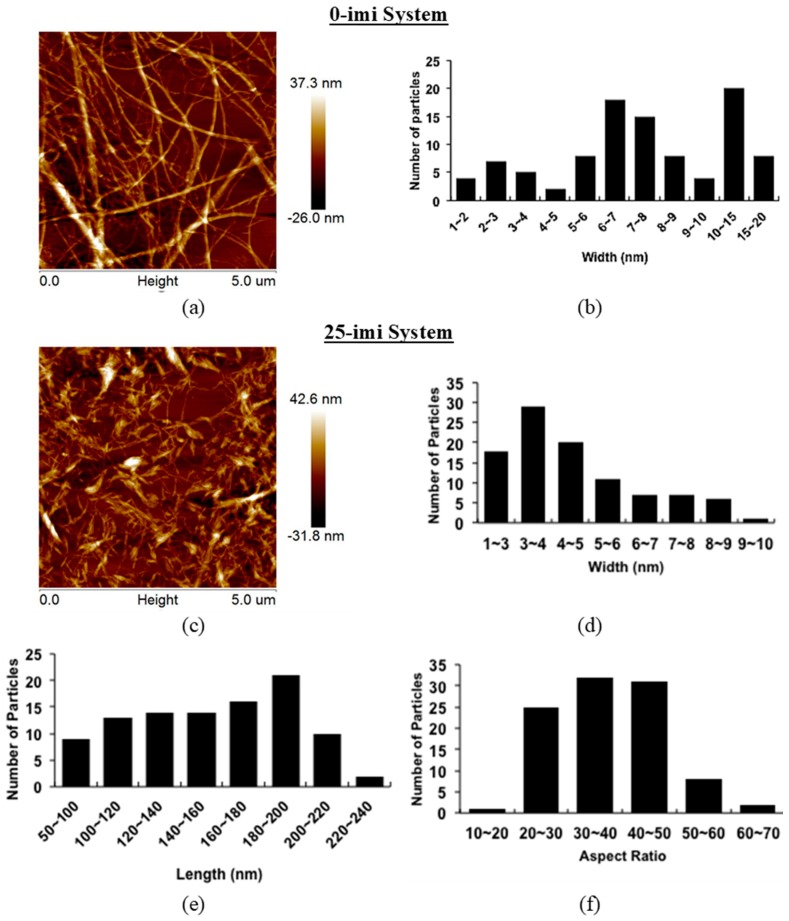
AFM height images and dimensional distributions evaluated from AFM images of CNFs obtained with pure imidazole (0-imi) system (**a**,**b**); and CNCs obtained with aqueous imidazole (25-imi) system (**c**–**f**).

The turbid suspensions with the highest yields, obtained with 0 and 25 wt % of water in imidazole, were selected for further characterization. Atomic force microscopy reveals the presence of CNFs when the pulp is treated with pure imidazole. Alternatively, CNCs are obtained when the pulp is treated with the imi-25 mixture (Figure 4a,c). CNFs exhibit an average width of ca. 8 ± 4 nm and a length of several micrometers (Figure 4a); CNCs display a width of 4 ± 2 nm and length of 156 ± 37 nm. The dimensional distributions including width, length and aspect ratio for CNCs are in the range of those obtained for conventionally obtained CNCs (Table 1). The CNFs, however, are more heterogeneous than CNFs obtained according to traditional mechanical or chemomechanical methods (15 nm or 3–5 nm [36,37]), with a width distribution ranging between 2 and 20 nm (Figure 4b). Molecular weight analysis of the obtained CNCs and CNFs supports the generation of CNFs and CNCs in absence or presence of water in imidazole, respectively. The number average degree of polymerization averages 604 after treating pulp with imidazole, in agreement with values reported for CNFs [38]. An average DP_n_ of 250 is computed for CNCs obtained by treating pulp with the imi-25 imidazole aqueous mixture.

**Figure 5:**
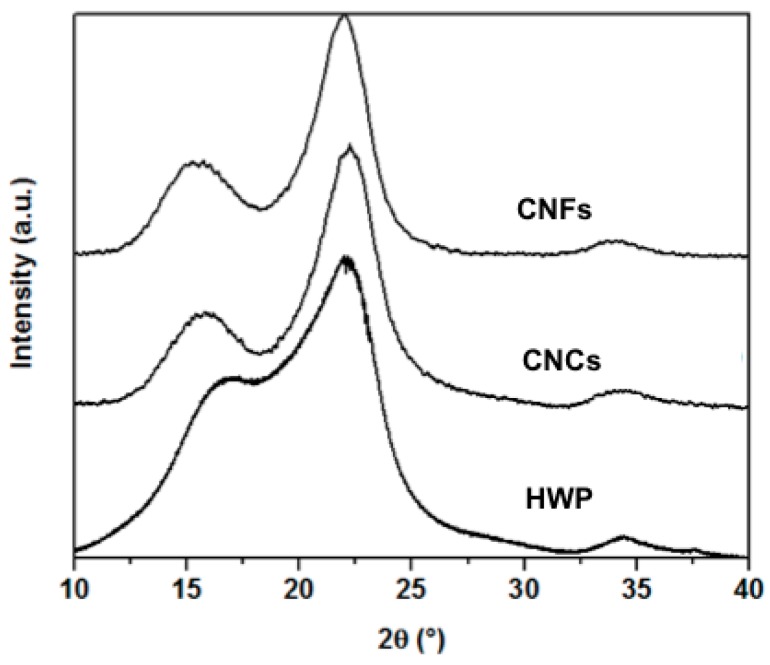
Wide angle X-ray diffraction (WAXD) patterns of the original bleached hardwood pulp (HWP), extracted CNFs and CNCs using 0-imi and 25-imi system, respectively.

To assess whether the native morphology of cellulose was preserved during the treatment, CNFs and CNCs were characterized by WAXD. For all samples, characteristic peaks of cellulose I appear at 2θ = 14.1°, 16.4°, 22.5° and 34.5°, which correspond to the crystallographic planes of [1–10], [110], [200] and [004], respectively (Figure 5). The native cellulose allomorph is thus retained in both types of nanocellulose (Figure 5). The coherence lengths of the crystallites in the thickness (τ) and length (B) directions are determined from the reflections [200] and [004] in raw HWP and both extracted nanocellulose samples. Both CNFs and CNCs exhibit similar τ and B of ca. 3 and 5 nm, respectively. These lateral dimensions are slightly smaller than those traditionally obtained for other CNFs or CNCs (Table 1). When comparing the crystallinity of all samples, similar crystallite thicknesses of both HWP (2.5 nm) and the extracted nanocellulose allow computing crystallinity indices according to the Segal method [34]. Expectedly, higher crystallinity indices of 67% and 73% are obtained for CNFs and CNCs, compared to the value of 43% for HWP (Table 1). These degrees of crystallinity are, however, significantly lower than those obtained according to traditional production methods of CNCs and CNFs (Table 1). Presumably, the imidazole treatment can be further optimized to improve crystalline recovery in nanocellulose.

### 3.3. Product Composition and Surface Analysis

Hardwood pulp, in general, contains higher amount of xylan (15–35%) compared to softwood pulp [39], and most of it can easily be dissolved during chemical or chemomechanical treatments. When reacting HWP containing ca. 12 wt % of xylan with imidazole, about 9 and 10 wt % of xylose remained in the produced CNFs and CNCs, respectively (Table 1). The contribution from xylan as an amorphous material might explain the lower CrIs for both nanocellulose types (Table 1). 

Xylan-rich nanofibrillated cellulose has been recently produced by TEMPO-mediated oxidation [40,41]. Their special core-shell structure with swollen soft polysaccharides on the surface of CNFs has shown interesting interfacial properties and rheological behavior, which differ from those of neat CNFs. Different xylan and carboxylate contents ranging between 9% and 25% and 0.8–1 mmol/g, respectively, could be adjusted by controlling the conditions, such as reaction time, oxidizing agent type and concentration, etc. [41]. The high-xylan CNFs produced in our work contained only approximately 40 μmol/g of carboxyl groups, which was much lower than the reported data for other CNFs using oxidizing reagents, such as a TEMPO-mediated system, with the similar amount of xylan [41]. The zeta-potential of imidazole liberated nanocelluloses reached ca. −30 mV. Those values were in the same range as those reported for TEMPO-fibrillated CNFs and IL-mediated CNCs, albeit not as high as those reported for CNCs obtained by sulfuric acid hydrolysis (Table 1) [41]. As a result the xylan-rich nanocellulose suspension could be electrically and sterically stabilized as also observed in SUP (Figure 2c) [42]. Although it is clear that the main form of hemicelluloses in hardwood pulp is glucuronoxylan [39], possible changes in the structure of xylan upon imidazole treatments, which can be concluded from the molar ratios of different monomers [43], are not yet clear.

Overall, these results indicate that imidazole and its aqueous mixtures are effective media for the production of nanocelluloses from pulp and that the imidazole concentration allows for tuning the process towards fibrillation with the production of CNFs (in pure imidazole) or towards the production of CNCs (in a 75/25 wt % imidazole/water mixture). Recent attempts to optimize yield have proved to be encouraging, with improvements up to ca. 40 wt % for CNFs. Process optimization and mechanistic studies will be the topic of a follow-up publication.

## 4. Conclusions

Treating hardwood pulp with imidazole liberates nanocelluloses, either as CNCs or CNFs, depending on the imidazole’s water content. With an aqueous imidazole system of 25 wt % water content, the DP_n_ of the treated pulp reached its lowest value and 20 wt % of CNCs were obtained; with pure imidazole, 10 wt % of CNFs were recovered. Both nanocelluloses displayed crystallinity indices on the order of 70%. This is lower than that typically reported for nanocellulose produced with conventional sulfuric acid hydrolysis and mechanical fibrillation methods. Interestingly, both types of nanocellulose also appeared to retain high xylan contents (ca. 10%) from the original hardwood pulp. This high xylan content likely explains the lower crystallinity index of the resulting nanocelluloses. At the same time, the presence of xylan in the final CNFs or CNCs products is expected to broaden their application field.

## Figures and Tables

**Table 1:** Characteristics of the original hardwood pulp (HWP), CNCs obtained using sulfuric acid (-SA), [Bmim]HSO_4_ (-IL), imidazole (-imi) and CNFs obtained using TEMPO-based system (-TEMPO) and imidazole (-imi).

Characterization		HWP	CNFs		CNCs		
			-imi	-TEMPO [44,45,46]	-imi	-SA [47]	-IL [19]
**Chemical Composition**	Xylose (%)	12.42 (2.02)	10.22 (1.54)	n/a	8.96 (2.45)	n/a	6.40 (0.96)
	Arabinose (%)	0.11 (0.01)	0.07 (0.01)	n/a	0.09 (0.02)	n/a	n/a
	4-*O*Me-GlcUA (%)	0.85 (0.00)	0	n/a	0	n/a	n/a
	Mannose (%)	0.11 (0.00)	0	n/a	0	n/a	0.18 (0.02)
**Morphologies**	W (nm)	10,000~20,000	8 ± 4	3~5, 15	4 ± 2	4.8 ± 0.4	6 ± 2
	L (nm)	>10,000	Several microns	Several microns	156 ± 37	147 ± 7	227 ± 74
	AR	~100	>100	>100	38 ± 10	20~30	43 ± 21
**Surface Properties**	* Zeta potential (mV)	‒10	‒26 ± 3	‒33	‒27 ± 5	‒50~‒60	‒20~‒30
	COOH (μmol/g)	50.0 (1.8)	40.3 (1.1)	0.1 × 10^3^~1.5 × 10^3^	44.5 (0.0)	n/a	n/a
	C=O (μmol/g)	12.9 (0.0)	30.6 (0.6)	10~100	32.1 (0.0)	n/a	n/a
**Crystallite Properties**	CrI (%)	54	67	65~95	73	70~90	77 ± 1
	Τ (nm)	4.2	3.3	4	2.9	4~6	4.4 ± 0.2
	B (nm)	5	5	6	5	6~30	6 ± 1
**Preparation Route**			Imidazole, filtration, sonication	TEMPO/NaBr/ NaClO, filtration	Imidazole/H_2_O, filtration, sonication	H_2_SO_4_, centrifugation, dialysis	[Bmim]HSO_4_, centrifugation, dialysis
**Yield (wt %)**			10	~90	20	20–30	~57

* Data was obtained at pH = 6.
